# ^1^H, ^13^C, and ^15^N NMR chemical shift assignment of LytM N-terminal domain (residues 26–184)

**DOI:** 10.1007/s12104-023-10151-5

**Published:** 2023-09-24

**Authors:** Ilona Pitkänen, Helena Tossavainen, Perttu Permi

**Affiliations:** 1 Department of Biological and Environmental Science, University of Jyvaskyla, FI-40014 Jyvaskyla, Finland; 2 Department of Chemistry, Nanoscience Center, University of Jyvaskyla, FI-40014 Jyvaskyla, Finland; 3 Institute of Biotechnology, Helsinki Institute of Life Science, University of Helsinki, FI-00014 Helsinki, Finland

**Keywords:** NMR spectroscopy, Ha-detection, LytM, *S. aureus*

## Abstract

Antibiotic resistance is a growing problem and a global threat for modern healthcare. New approaches complementing the traditional antibiotic drugs are urgently needed to secure the ability to treat bacterial infections also in the future. Among the promising alternatives are bacteriolytic enzymes, such as the cell wall degrading peptidoglycan hydrolases. *Staphylococcus aureus* LytM, a Zn^2+^-dependent glycyl-glycine endopeptidase of the M23 family, is one of the peptidoglycan hydrolases. It has a specificity towards staphylococcal peptidoglycan, making it an interesting target for antimicrobial studies. LytM hydrolyses the cell wall of *S. aureus*, a common pathogen with multi-resistant strains that are difficult to treat, such as the methicillin-resistant *S. aureus*, MRSA. Here we report the ^1^H, ^15^N and ^13^C chemical shift assignments of *S. aureus* LytM N-terminal domain and linker region, residues 26–184. These resonance assignments can provide the basis for further studies such as elucidation of structure and interactions.

## Biological context

The increasing prevalence of antimicrobial resistance in bacteria is among the most pressing threats to public health (Murray et al. 2022). The higher mortality and morbidity associated with antibiotic resistant bacterial infections burden the operation of healthcare as well as impose significant economic and societal costs (World Health Organization 2014). The background of the problem is multifaceted, stemming from the overuse and misuse of current antimicrobial drugs combined with the scarcity of new prominent antibiotics (Gould and Bal 2013; Ventola 2015). Thus, development of alternative solutions is urgently needed to avoid a future in which antibiotic drugs are no longer an effective treatment for bacterial infections.

One of the most problematic bacterial species is *Staphylococcus aureus*, a major human pathogen causing a wide variety of infections of different severity. *S. aureus* is acknowledged as the leading cause in skin and soft tissue infections, particularly infections occurring in surgical sites and associated with medical implants (Lowy 1998; Tong et al. 2015; Diekema et al. 2019). *S. aureus* has several features which make it a challenging pathogen to treat, and among them is the capability to rapidly acquire resistance to antimicrobial drugs. Conformingly, the methicillin-resistant *S. aureus* (MRSA) is reported to have gained resistance against many of the first-line antibiotics, including the traditional β-lactam antibiotics (Monaco et al. 2017). Therefore, it is particularly alarming that an emerging resistance to last-resort antibiotics such as vancomycin has been observed in MRSA strains, making MRSA one of the most serious infectious disease threats globally (Guo et al. 2020).

Among the promising approaches to combat antibiotic resistant strains of bacteria is the utilization of bacteriolytic enzymes, such as the peptidoglycan hydrolases (PGHs). PGHs kill bacteria via degradation of peptidoglycan, an essential component of the bacterial cell wall (Szweda et al. 2012). PGHs have multiple beneficial attributes, including their efficacy against biofilms and the low likelihood of inducing the development of resistance in bacteria due to their specificity and the conserved nature of their target, the peptidoglycan (Pastagia et al. 2013).

LytM is a Zn^2+^-dependent glycyl-glycine endopeptidase belonging to the M23 family of metallopeptidases. The target of its catalytic activity is the pentaglycine bridge specific for *S. aureus* peptidoglycan, making it an interesting candidate for treating *S. aureus* infections. The structure of this enzyme has been studied by the means of X-ray crystallography, yielding a two-domain structure (Odintsov et al. 2004). The C-terminal domain with a conserved catalytic site harbours the catalytic activity of the enzyme, whereas the function of the N-terminal domain is currently unknown. The unique N-terminal domain sets LytM apart from the other enzymes of the same family, such as *S. simulans* lysostaphin (Sabala et al. 2014; Tossavainen et al. 2018) and *S*. *aureus* LytU (Raulinaitis et al. 2017). LytM has been reported to be inactive in its full form, but the catalytic domain alone has been shown to cleave peptidoglycan (Odintsov et al. 2004). The activation mechanism of LytM, however, remains unclear. In the latent form the catalytic site is blocked by an occluding residue Asn117 residing in the linker, and consequently an “asparagine switch”, analogous to the cysteine switch of matrix metalloproteases, has been proposed for the regulation of the activity (Odintsov et al. 2004). This highlights the importance of understanding the role of the N-terminal domain and the linker region in the activation. To this end, we have characterized the LytM N-terminal domain and linker region by assigning the chemical shifts of residues 26–184, including the previously uncharacterized, potentially disordered regions in the crystal structure (Odintsov et al. 2004).

## Methods and experiments

### Protein expression and purification

Two constructs of *S. aureus* LytM, including the N-terminal domain (residues 26–107) and N-terminal domain with the linker region (residues 26–184) were cloned in pET-15b vectors. To improve protein solubility the LytM constructs were tagged with GB1 in the N-terminus connected with linker region containing the TEV protease site. Proteins were expressed using *Escherichia coli* strain BL21(DE3). 50 ml precultures were grown in standard M9 supplemented with 100 µg/ml ampicillin at 30 °C with 230 rpm shaking for 20 h. Cultures were expanded to 1 l and the cells were grown at 37 °C with 250 rpm shaking in standard M9 minimal medium supplemented with 100 µg/ml ampicillin and ^15^NH_4_Cl (1 g/l) and ^13^ C-D-glucose (2 g/l) as the nitrogen and carbon sources for uniform ^15^N and ^13^C labelling, respectively, until the OD at 600 nm was 0.55–0.6. Cells were cooled down to 16 °C, and the protein expression was induced by adding 0.5 mM isopropyl β-D-1-thiogalactopyranoside (IPTG) at 16 °C for 20 h, at 230 rpm.

Cells were harvested by centrifugation, resuspended in 50 mM sodium phosphate pH 8, 300 mM NaCl buffer and lysed by sonication (three times 1 min, 1 s pulse, 30% amplitude). Clarified lysates were purified with 1 ml His GraviTrap (Cytiva) column according to manufacturer’s instructions. GB1 was cleaved using TEV protease, overnight incubation in dialysis (buffer 25 mM sodium phosphate pH 8, 150 mM NaCl, 10% glycerol, 1 mM β-mercaptoethanol) at 4 °C. Cleaved proteins were purified in 20 mM sodium phosphate pH 6.5, 50 mM NaCl, 1 mM DTT buffer by size exclusion chromatography using ÄKTA pure chromatography system (GE Healthcare) with HiLoad Superdex S75 (16/60) column (GE Healthcare). Proteins were concentrated using Amicon Ultra-15 centrifugal filter units (Millipore).

### NMR spectroscopy

The NMR data acquisition was done using uniformly ^15^N,^13^C labelled LytM(26–107) and LytM(26–184) protein fragments in a buffer composed of 20 mM sodium phosphate, 50 mM NaCl, 1 mM DTT, pH 6.5 in 95/5% H_2_O/ D_2_O. The concentrations of LytM(26–107) and LytM(26–184) used for the data collection were 1.0 mM and 0.2 mM, respectively. NMR spectra were acquired at 308 K using a Bruker Avance III HD 800 MHz NMR spectrometer equipped with a ^1^H, ^13^C, ^15^N TCI cryoprobe.

Full chemical shift assignment was performed for LytM(26–107). Backbone resonances were assigned using ^1^H,^15^N HSQC (Kay et al. 1992) and ^1^H,^13^C-CT-HSQC (Vuister and Bax 1992) experiments for aliphatic and aromatic regions, and a suite of triple resonance NMR experiments including HNCO (Muhandiram and Kay 1994) and i(HCA)CO(CA)NH aka iHNCO (Mäntylahti et al. 2009), and HNCACB and HN(CO)CACB (Yamazaki et al. 1994). Aliphatic side chain resonances were assigned using H(CC)(CO)NH, (H)CC(CO)NH, HBHA(CO)NH and HCCH-COSY, whereas aromatic side chains were assigned using (HB)CB(CGCD)HD, (HB)CB(CGCDCE)HE and NOESY-^13^C-HSQC for aliphatic and aromatic carbons (Sattler et al. 1999).

The Hα, Cα, N and C′ chemical shifts of the LytM linker region (residues 108–184) were assigned using the longer construct LytM(26–184) and by employing Hα-detected 4D HACANCOi and HACACON (Tossavainen et al. 2020; Karjalainen et al. 2020), 3D HA(CA)NCOi (Karjalainen et al. 2020) and HA(CA)CON (Mäntylahti et al. 2010), together with HNCO and iHNCO experiments.

The NMR data were processed with TopSpin 3.5 pl 7 (Bruker Corporation) and analysed using CcpNmr Analysis v.2.4.2 software (Vranken et al. 2005).

## Extent of assignment and data deposition

**Fig. 1:**
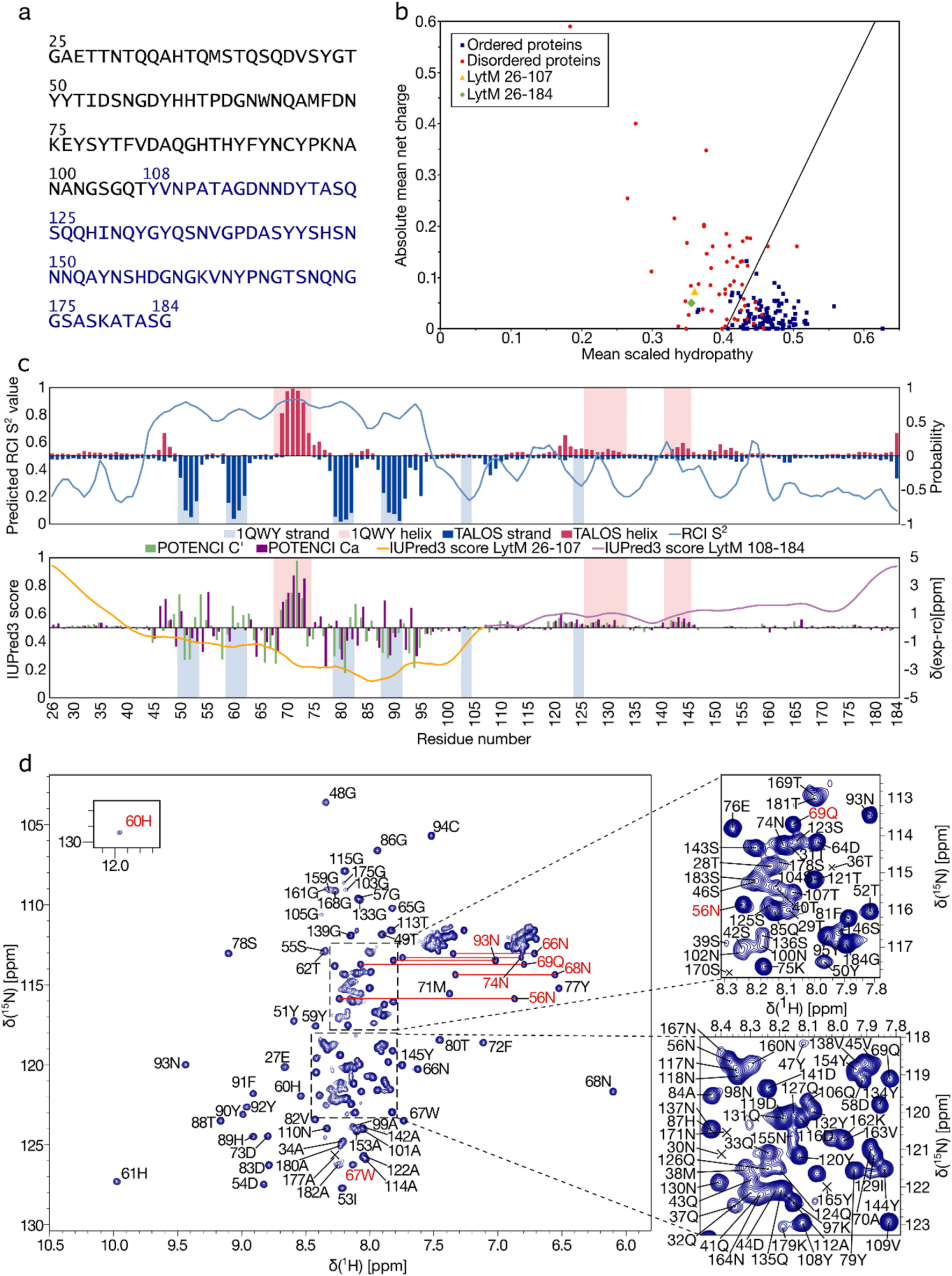
**a** The amino acid sequence of LytM N-terminal domain and linker region. The N-terminal region (26–107) is marked in black and the linker region (108–184) is marked in blue. The N-terminal Gly is a cloning artefact, and not a part of the native sequence. **b** The charge-hydropathy plot of LytM N-terminal domain and linker region, showing LytM(26–107) (yellow triangle) and LytM(26–184) (green diamond) compared to a set of ordered (blue squares) and disordered (red circles) proteins. **c** Results of structural analyses of LytM N-terminal domain and linker region, plotted against the residue numbers. The upper part of the panel shows TALOS-N predictions. The predicted RCI S^2^ value is plotted as a line chart (axis on the left) and the probabilities of helical (in red, as positive values) and strand (blue, negative) secondary structures are plotted in the predicted secondary structure bar chart (axis on the right). The lower part is a combined graph of IUPred3 prediction of intrinsic disorder (line chart, axis on the left) and secondary structure prediction (bar chart, axis on the right). In the IUPred3 prediction, the probability of disorder is presented as a score from 0 to 1, with higher value indicating higher propensity for disorder. LytM N-terminal domain (26–107) is marked with a yellow line, the linker region (108–184) with a light purple line. The secondary structure prediction is based on the difference in Cα (dark purple bars) and C’ (green) chemical shift values and random coil shifts generated by POTENCI. Positive values denote helical structures and negative values strand structures. Secondary structure elements observed in the crystal structure of full-length LytM (PDB ID 1QWY) are highlighted on both sections, beta strands in light blue and alpha helices in pink. **d** Assigned ^1^H,^15^N HSQC spectrum of LytM N-terminal construct with full linker (26–184), acquired at 308 K and 800 ^1^H MHz frequency, showing one-letter codes and residue numbers. Amides with peaks at contour levels lower than shown here are marked with crosses. Side chain assignments for Asn δ2, Gln ε2, His ε2 and Trp ε1 are marked in red. Asn δ2 and Gln ε2 side chain resonances are connected with horizontal lines. Insets on the right show the enlarged view of the areas indicated with dashed rectangles, encompassing the crowded regions. Inset on the left shows an additional peak outside the shown area

The amino acid sequence of LytM N-terminal domain and linker region, shown in Fig. 1a, was analyzed using bioinformatical tools to evaluate intrinsic disorder. A mean charge against mean scaled hydropathy plot (Fig. 1b), based on a PONDR (Xue et al. 2010) prediction, categorized both LytM N-terminal domain and N-terminal domain with linker region among intrinsically disordered proteins. For further evaluation, an IUPred3 (Erdos et al. 2021) analysis describing the probability of disorder within the sequence was performed. The results (Fig. 1c) show that most of the sequence has an IUPred3 score higher than 0.5 and is thus expected to be disordered. When only the N-terminal domain (yellow in Fig. 1c) is considered, the N- and C-terminal regions appear disordered, whereas the middle part scores lower values. Residues 41–106 have scores below 0.5, indicating ordered structure. The entire linker region (light purple in Fig. 1c) appears disordered, with the lowest probability for disorder being found locally around residue Val138.

The conclusion of intrinsic disorder is supported also by the experimental data. An overview of the ^1^H,^15^N HSQC spectrum of LytM(26–184) (Fig. 1d) shows that while some of the cross-peaks are well-dispersed in the ^1^H^N^ dimension, the majority are located between 7.5 and 8.5 ppm. All the well-dispersed correlations arise from the middle part of the N-terminal domain, particularly residues 51–94. The peaks assigned to the linker region (108–184), on the other hand, are all found within 7.8–8.3 ppm in the ^1^H^N^ dimension, and the signals overlap considerably. This discrepancy in the distribution of peaks between the two regions of the protein was also reflected in the extent of chemical shift assignment.

The chemical shifts of the N-terminal domain (residues 26–107) were assigned more comprehensively. The backbone chemical shifts were assigned nearly completely, with an assignment percentage of 98.8%. Out of the non-proline residues, 97.5% of ^1^H-^15^N pairs were assigned, omitting the N-terminal glycine not belonging to the native sequence. 98.8% of C′ and all Cα, Hα, Cβ and Hβ resonances were assigned. The aliphatic side chain assignment was 73.6% complete, and aromatic side chain assignment completeness was 93.2%. The low aliphatic side chain assignment percentage was largely due to the unassigned Asn δ2 (55.6% assigned) and Gln ε2 (12.6%) resonances. These resonances could not be reliably identified due to overlap of signals in the ^1^H,^15^N HSQC spectrum.

**Fig. 2:**
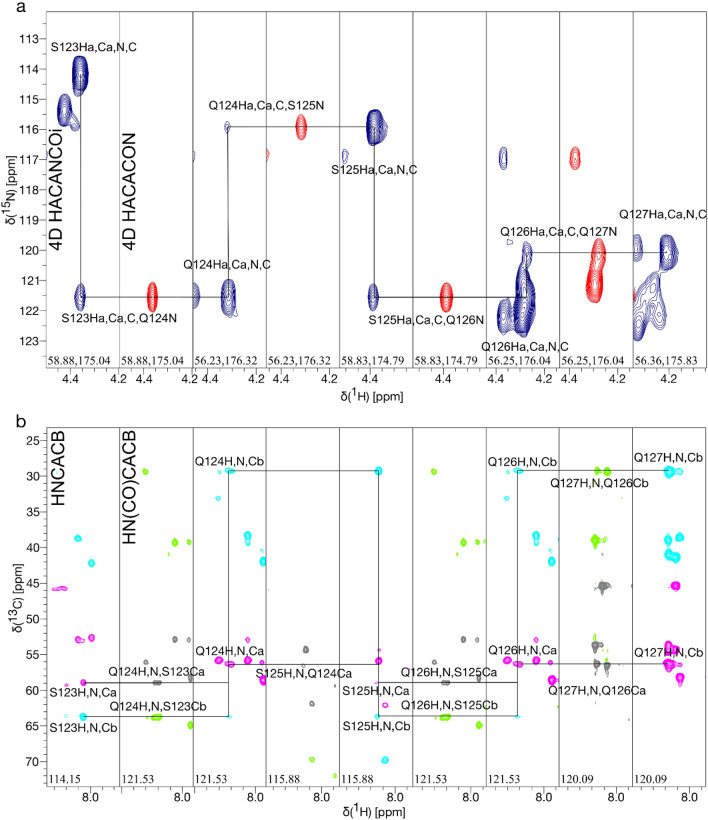
Strip plots showing the sequential walk through residues 123-127 of LytM using **a** 4D HACANCOi (blue) and HACACON (red) experiments and **b** 3D HNCACB (Cα magenta, Cβ cyan) and HN(CO)CACB (Cα grey, Cβ green) experiments. The spectra of 4D experiments are less crowded and no peaks are missing, unlike in the 3D experiments

The backbone chemical shifts of the disordered linker region (residues 108–184) were assigned using additional 3D HA(CA)NCOi and HA(CA)CON, and 4D HACANCOi and HACACON experiments to overcome the issue of signal overlap and missing amides in the ^1^H,^15^N HSQC spectrum. The process is described in Fig. 2. The assignment percentage for the backbone of the complete LytM N-terminal domain with the linker region was 94.9%. The missing resonances were mainly amides, including residues Ala26, His35, Pro63, Asn151 and His157, emphasizing the decision to opt for HA-detected experiments. Additionally, assignment percentage was affected by C’ missing from 46Ser, and Cα from His147 and Ser156. Residues Asn151 and His157 remained completely unassigned.

These data have been deposited in BioMagResBank (www.bmrb.wisc.edu) under accession number 51662.

For further characterization, the chemical shifts were used to investigate the secondary structure content of LytM N-terminal domain and linker region. Two approaches, TALOS-N (Shen and Bax 2013) and secondary structure prediction based on the deviations of the chemical shifts of Cα and C′ from random coil chemical shifts predicted by the POTENCI (Nielsen and Mulder 2018) tool are shown in the upper and lower halves of Fig. 1c, respectively. Similar results were obtained from both analyses, showing that the N-terminal domain has indications for secondary structure mainly on residues 45–95. This conclusion is reciprocated also in the results of IUPred3 analysis and predicted random coil index order parameter (RCI S^2^) values derived from TALOS-N. These analyses outline a similar increase of order and decrease of flexibility in the mid-N-terminal domain.

Comparison with the secondary structure elements of the crystal structure of full-length LytM (PDB ID 1QWY, Odintsov et al. 2004) agrees well with these findings (Fig. 1c). The alpha helix at residues 68–74 is detected in both secondary structure predictions but TALOS-N appears more sensitive for the beta strand elements in the N-terminal domain. On the other hand, residues 26–44 which are missing from the crystal structure show less propensity for secondary structure in the predictions, conforming to the expectation. Similar observation is made regarding the linker (108–184), out of which residues 147–182 are not defined in the crystal structure. When the characterized residues 108–146 are considered, they appear mostly disordered with the exceptions of transient helical structures that are observed in residues 126–133 and 141–145, and propensity to extended structure in residues 124–125. Both secondary structure predictions agree on the disordered nature of the linker region. Helical elements can be detected in both analyses; based on the results at least residues 120–131 and 142–146 would be expected to show transient helical structure. The presence of the transient helical elements in the truncated protein indicates that their structure is not completely dependent on the interaction with the catalytic domain. The beta strand structure of residues 124–125, on the other hand, does not show clear pattern in the prediction, suggesting that it is formed through the interaction.

Here we have presented the chemical shift assignment of LytM residues 26–184. A nearly complete assignment was obtained for N-terminal residues 26–107, which are likely to form a structured domain. Based on chemical shift dispersion and bioinformatics, residues downstream 107 are mainly disordered. Hα, HN, Cα, Cβ, C, and NH resonances were assigned for these residues. These assignments allow further studies such as structural characterization and investigation of interactions.

## Data Availability

The chemical shifts have been deposited in BioMagResBank (www.bmrb.wisc.edu) under Accession Number 51662.
